# Dynamic monitoring of cell mechanical properties using profile microindentation

**DOI:** 10.1038/srep21529

**Published:** 2016-02-09

**Authors:** L. Guillou, A. Babataheri, P.-H. Puech, A. I. Barakat, J. Husson

**Affiliations:** 1 Hydrodynamics Laboratory (LadHyX), Department of Mechanics, Ecole Polytechnique, 91128 Palaiseau, France; 2 Aix Marseille University, LAI UM 61, Marseille, F-13288, France; 3 Inserm, UMR_S 1067, Marseille, F-13288, France; 4 CNRS, UMR 7333, Marseille, F-13288, France

## Abstract

We have developed a simple and relatively inexpensive system to visualize adherent cells in profile while measuring their mechanical properties using microindentation. The setup allows simultaneous control of cell microenvironment by introducing a micropipette for the delivery of soluble factors or other cell types. We validate this technique against atomic force microscopy measurements and, as a proof of concept, measure the viscoelastic properties of vascular endothelial cells in terms of an apparent stiffness and a dimensionless parameter that describes stress relaxation. Furthermore, we use this technique to monitor the time evolution of these mechanical properties as the cells’ actin is depolymerized using cytochalasin-D.

In cells, the cytoskeleton is a key determinant of mechanical properties. Therefore, biological processes that involve extensive cytoskeletal remodeling such as cell division, differentiation, and migration have been shown to be associated with changes in cell mechanical properties^1^,^2^. Cell responses to chemical and biophysical cues in their microenvironment also often lead to structural changes that impact mechanical properties^3^,^4^,^5^,^6^,^7^. For instance, during inflammation, leukocyte-endothelial cell interactions affect the mechanical properties of both cell types, which can in turn affect transmigration^5^,^8^. There is, therefore, great interest in measuring the evolution of cell mechanical properties over time as a way of monitoring structural and functional changes that cells undergo during key biological processes. Furthermore, mechanical forces play a key role in the development of major diseases. For instance, the mechanical properties of tissues contribute in some cases to cancer progression and may also affect treatment outcome^9^.

Several techniques have been developed to probe cell mechanical behavior. These include micropipette aspiration^10^,^11^, atomic force microscopy (AFM)^12^,^13^,^14^,^15^,^16^,^17^,^18^, micro-plates^19^,^20^, optical tweezers^21^,^22^, magnetic twisting cytometry^21^,^23^, particle tracking^24^,^25^, and microfluidic cell stretchers^1^,^2^. These various techniques have been used to probe either local (cortex, cytoplasm, etc.) or whole-cell mechanical properties at different spatial and time scales.

In this paper, we introduce profile microindentation (PM) as a simple and minimally disruptive method for assessing viscoelastic properties at a single-cell level. PM involves using a microindenter to indent a cell while using brightfield imaging from profile both to visualize the cell and to determine the deflection of the microindenter. The measurements can be made sufficiently rapidly (~10 s) to monitor the evolution of cell mechanical properties at biologically relevant time scales (few minutes to several hours). During the measurements, cell deformations are directly visible, offering a view of the cell that has seldom been reported^15^. Furthermore, we can readily add another micropipette to the setup to stimulate the cell locally in a highly controlled manner. This micropipette can, for instance, be used to “whiff” a drug or another chemical onto the cell, to bring another cell or micron-sized object such as an antibody-covered microbead in contact with the cell, or to directly stimulate the cell mechanically through aspiration or indentation. To demonstrate this capability, we “whiffed” cytochalasin-D onto bovine aortic endothelial cells (BAECs) and measured the evolution of their mechanical properties over a period of more than 30 minutes. A limitation that this technique shares with many other systems that probe cell mechanical behavior is its relatively low throughput.

## Methods

### Endothelial cell culture

BAECs were kindly provided by A.-C. Vion and C. Boulanger and used between passages 4 and 12. The cells were cultured at 37 °C and 5% CO_2_ in Dulbecco’s Modified Eagle’s medium (DMEM, Invitrogen, Carlsbad, CA, USA) supplemented with 10% fetal bovine serum (Invitrogen) and 1% penicillin/streptomycin (Invitrogen). The cells were passaged two to three times a week and re-suspended in fresh culture medium. One to two days before each experiment, the cells were trypsinized with trypLE (Invitrogen) and grown on Cytodex-3 dextran microcarrier beads (average bead size 175 μm, GE Healthcare Life Sciences, Velizy-Villacoublay, France). For the experiments, about 50 Cytodex-3 beads without cells were deposited onto the bottom of a thin-bottom petri dish (standard bottom μ-Dish 35 mm low, IBIDI, Martinsried, Germany or FluoroDish 35 mm, World Precision Instruments, Hitchin, UK) in phosphate buffered saline (PBS; Invitrogen). The PBS was then removed and ~10,000 trypsinized BAECs were introduced into the petri dish.

Human umbilical vein endothelial cells (HUVECs) for the profile microindentation experiments were kindly provided by A. Chipont, and originally purchased from PromoCell (PromoCell GmbH, Heidelberg, Germany). The cells were cultured at 37 °C and 5% CO_2_ in endothelial cell growth medium (ECGM) procured from ZenBio (ZenBio, Research Triangle Park, North Carolina, USA). The protocol for depositing on Cytodex-3 beads differed from that used for BAECs in that after mixing cells and beads and letting them rest in the incubator for 30 min in a 2 mL eppendorf tube (Eppendorf France SAS, Montesson, France), the tube was placed for 3 hours on a rotating plate turning at 50 rpm and heated at 37 °C to ensure optimal coverage of the beads. HUVECs used for the AFM experiments were obtained from PromoCell (ref. C-12203) and cultured according to the supplier’s guidelines using ECGM-2 medium (ref. C-22011). Trypsin/EDTA was used for cell passaging.

While most experiments were performed at room temperature, we verified that our method could also be employed at physiological temperature (see supplementary discussion). In some experiments, cells were exposed to cytochalasin-D (Sigma-Aldrich, Taufkirchen, Germany) either by incubation or by “whiffing” the drug onto cells with a micropipette.

### Microscope setup

In all microindentation experiments, the petri dish containing cells on Cytodex-3 beads was mounted on a TE300 inverted microscope (Nikon Instruments, Tokyo, Japan) placed on an air suspension table (CVI Melles Griot, Netherlands). The microscope was equipped with a 100× oil immersion, 1.3 NA objective (Nikon Instruments) for experiment monitoring and lower magnification objectives (40×, 20×, 10×, and 4×, Nikon) for micropipette positioning. Images were acquired using a Flash 4.0 CMOS camera (Hamamatsu Photonics, Hamamatsu City, Japan) controlled using the software LabVIEW (National Instruments, Austin, TX, USA). We will provide the LabVIEW codes upon request.The experiments were performed using either brightfield or fluorescence microscopy. Supplementary movie S1 shows a demonstration video of profile microindentation.

### Micropipette and microindenter fabrication

Borosilicate glass capillaries (1 mm OD, 0.78 mm ID, Harvard Apparatus, Holliston, MA, USA) were pulled on a P-97 micropipette puller (Sutter Instruments, Novato, CA, USA). To fabricate the micropipettes, an MF-900 microforge (Narishige, Tokyo, Japan) was used to cut the extremity of pulled capillaries to the desired diameter, ranging from ~4 to 50 μm. The diameter was assessed optically using calibrated graduations in the microscope’s ocular. The micropipettes were then bent at a 45° angle (for the micropipette holding the Cytodex-3 bead) or a 60° angle (for the micropipette “whiffing” the drug) so that their extremities had the desired direction in the microscope’s plane of view. To fabricate a microindenter, an MF-200 microforge (World Precision Instruments) was used to melt glass at the tip of the micropipette. During fabrication, using graduations in the microscope’s ocular, we aimed for indenter tips that were 5 to 10 μm in diameter. The size was then precisely determined under the inverted microscope using the 100× objective. The microindenter’s bending stiffness was evaluated against standard microindenters that had been previously calibrated. The standard microindenters were calibrated by measuring their deflection under the gravitational force exerted on their tip by a piece of paper of known mass. While microindenters made for these experiments were typically of rigidities ~5 to 10 nN/μm, their rigidity can be chosen as they are custom-made. Microindenters of rigidities as low as 0.1 nN/μm are routinely used in our laboratory for measurement of sub-nanonewton forces.

### Micromanipulators and piezoelectric controller

The experimental setup was equipped with two motorized micromanipulators (MP285, Sutter Instruments) carrying two micropipette holders (IM-H1, Narishige) at a 45° angle (different angle from the micropipette bending). One micropipette was used to hold Cytodex-3 beads, while the other one was used to “whiff” the drug onto the cell. A piezoelectric controller (TPZ001, Thorlabs, Newton, NJ, USA) along with a strain gauge reader (TSG001, Thorlabs) were used to control the microindenter. Because profile microindentation requires only a single-axis piezoelectric, a micromanipulator (or two if another micropipette is introduced), a camera able to acquire images at 30 Hz, and a high magnification objective, it is a technique that is relatively low-cost and simple to implement.

### Actin visualization

To visualize intracellular actin filaments in living cells, BAECs were transfected with the live-cell actin marker LifeAct. A day before transfection, cells were plated on a 35 mm-diameter FluoroDish (World Precision Instruments) at densities that led to 50–80% confluence the following day. For transfection, cells were incubated at 37 °C in a mixture of 200 μL cell culture medium, 2 μg LifeAct DNA (pIRES-LifeAct-GFP-puro3, IBIDI), and 8 μL GeneCellin (DNA Transfection Reagent, BioCellChallenge, Signes, France) for a period of 24 h. The cells were then washed with new medium before imaging. For fluorescence excitation, an Intensilight (C-HGFIE, Nikon) lamp with GFP illumination was used.

### Single-cell profile microindentation

**Figure 1:**
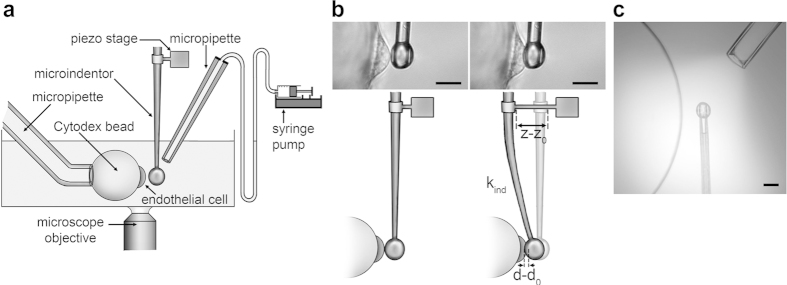
Experimental setup for profile microindentation. (**a**) Schematic (not drawn to scale) of the experimental setup used for profile microindentation. Microindenter is used to exert force on the endothelial cell adherent at the equator of the Cytodex-3 bead. (**b**) Microindenter before (left) and during (right) cell indentation. Scale bar is 10 μm. (**c**) Photograph of fluorescein “whiffed” by the micropipette on the Cytodex-3 bead in order to visualize the convection cone coming out of the micropipette. Scale bar is 10 μm.

Culturing cells on Cytodex-3 beads allowed us to image the cells in profile, which permitted visualization of cell deformation upon indentation. Cells were indented above the nucleus. During cell indentation, the Cytodex-3 bead was held in place using a micropipette with an aspiration pressure as shown in Fig. 1. Using the piezoelectric controller, we applied a known displacement z to the base of the glass microindenter, with the piezoelectric controller moving at a constant speed v. We monitored the position d of the microindenter’s spherical tip using an algorithm running in real-time in LabVIEW that cross-correlated the brightness profile of the current image with the brightness profile of the initial image before indentation, as already used by Husson *et al.*^26^ and Laan *et al.*^27^. This correlation was performed over a rectangular region of interest, and brightness was averaged over 4 pixels. Because we fit an entire region rather than a single pixel, our spatial resolution is smaller than the pixel size (60 nm/px at 100× magnification). Including the effect of ambient noise, we found that the standard deviation on the position of an indenter at rest was typically 30–40 nm. Contact between the microindenter and the cell was indicated by an increase in the indenter’s deflection (d–z) compared to its initial value before any piezoelectric controller movement (d_0_ − z_0_). Because the indenter’s deformation remained small during the indentation (deformation ~deflection/length ~0.01), the applied force F was linearly related to the deflection through the indenter’s stiffness F = k_ind_ [(d − z) − (d_0_ − z_0_)]. We continued the indentation until we reached a previously selected threshold force F_threshold_. We recorded the tip’s position d_max_ (typically 1 to 2 μm indentation, which is approximately 20% of the cell thickness above the nucleus) at this point in time and then used a feedback loop to adjust in real-time the displacement z imposed by the piezoelectric controller to maintain that position constant as the cell relaxed. Thus, after the approach phase, the strain was maintained constant throughout the relaxation phase. We let the cell relax for at least 10 s before retracting the indenter. Data acquisition frequency was ~30 Hz. Thus, for data analysis, we had access to the force applied by the microindenter F, the tip’s position d, the imposed piezoelectric controller movement z, and the time t of each measurement.

### Atomic Force Microscopy

Adherent cells were cultured on 70% ethanol-cleaned glass slides in 6-wells culture plates, rinsed to remove unbound cells and fragments and mounted in a temperature-controlled chamber (Biocell, JPK Instruments, Berlin, Germany) set to 37 °C. Cells were indented with a JPK Nanowizard 1 AFM (JPK Instruments), using the force mode with a closed loop 15 μm range piezo. The AFM sits on an Axiovert 200 microscope equipped with a Colibri 2 diode illumination system (Zeiss, Oberkochen, Germany) and a CoolSnap HQ2 camera (Photometrics, Tucson, AZ, USA). A glass sphere of diameter 10 μm was glued by micromanipulation (using a homemade micropipette/biomembrane force probe setup) to a gold-coated triangle-shaped MLCT cantilever (Bruker Instruments, Billerica, MA, USA), using UV polymerizable glue (Dymax OP-29) in order to measure cell mechanics on similar scales as in the microindentation experiments. The decorated AFM cantilever was calibrated *in situ* prior to the experiments using the thermal noise method implemented in the JPK SPM control software and found to be 11.5 nN/μm, compatible with the nominal data provided by the manufacturer (10 nN/μm). The approach and retract speeds of the indenter were 1 μm/s over a distance of 5 μm and the maximal applied force was set between 3 and 6 nN. The acquisition frequency was set at 1024 Hz.

### Data and statistical analysis

Raw data acquired by LabVIEW were analyzed using a custom-written code in MATLAB (The MathWorks, Natick, MA, USA). We will provide the code upon request. AFM data were processed using JPK DP software (JPK Instruments) using built-in fitting procedures. Statistical comparisons between two groups were performed using the two-tailed Student t-test. Tests were unpaired unless otherwise noted. Statistical comparisons among three groups or more were performed using a one-way ANOVA test. Statistical comparisons between slopes were performed using an ANOCOVA test. Samples were deemed statistically significantly different for p < 0.05.

## Results

### Precision of displacement and force measurement in profile microindentation

**Figure 2:**
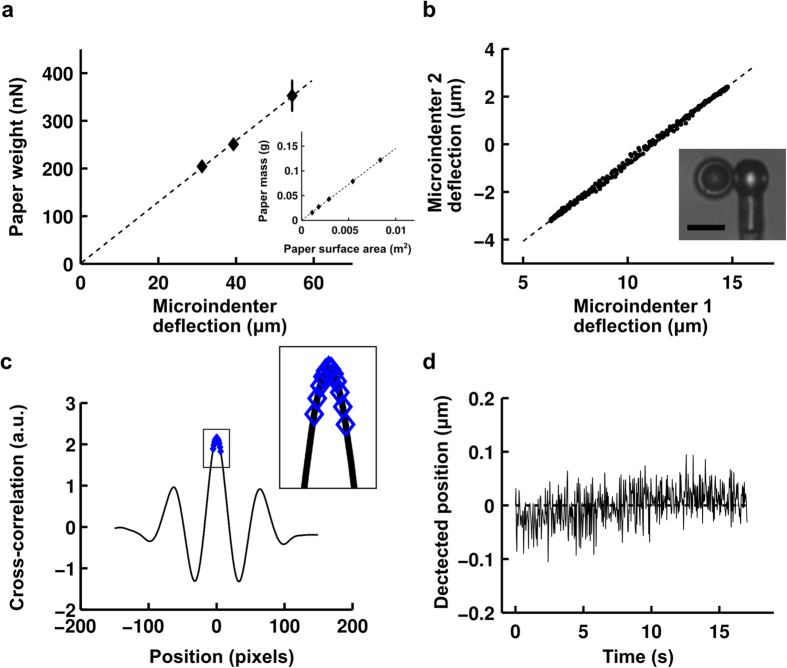
Profile microindentation calibration and noise level. (**a**) To calibrate a reference microindenter, its deflection under the weight of pieces of dry paper of known mass is measured. Data are mean ± s.e.m. Papers adhere to the tip by dipping the tip in oil. The inset shows how paper density was ascertained by measuring the masses of pieces of paper whose surface areas were then measured under the microscope. (**b**) Microindenters used in the experiments were calibrated against reference microindenters by measuring the ratio of their deflections when pushed against one another. Scale bar is 10 μm. (**c**) A profile of the light intensity was measured on a line along the axis of indentation, and a template profile for the image of the indenter on that line indenter shaft was taken. A parabolic fit over 10 pixels (blue diamonds, see inset) was used to find the maximum of the cross-correlation curve, giving the position with sub-pixel resolution (~30 nm, see panel **d**). (**d**) The position of the indenter was measured at rest over a period of ~15 s, comparable to the time of stress relaxation experiments performed, to evaluate the combined error stemming from noise in the environment and measurement error. At an acquisition frequency of ~30 Hz, the standard deviation of the position is 32 nm in the representative data shown.

The first step in the microindentation experiments is to calibrate the microindenters. We first determined precisely the density of a type of paper by measuring the mass of pieces of this paper whose surface area was then measured under the microscope (Fig. 2). We then calibrated reference microindenters by measuring their deflection under known weights of pieces of paper. The results demonstrated that we remain in the linear elastic regime for the range of deformations tested. Microindenters used in the experiments were calibrated against the reference microindenters by measuring their deflections when pressed against each other (Fig. 2). The ratio of the deflections of the two microindenters directly provides the ratio of their rigidities.

To measure the position of the microindenters during profile microindentation, we acquire a profile of the light intensity on a line along the axis of indentation at a frequency of ~30 Hz. This intensity profile is compared using cross-correlation against a template profile for the image of the indenter. A parabolic fit over 10 pixels is then used to find the maximum of the cross-correlation curve (Fig. 2), giving the position of the indenter.

At a magnification of 100×, the size of a pixel is ~60 nm. However, the parabolic fit used here allows sub-pixel resolution. Including the noise in the environment and at an acquisition frequency of ~30 Hz, we find on a typical day that we are able to determine the position of the indenter with a precision of half a pixel, or about ~30 nm (Fig. 2). For a typical indenter of rigidity 5–10 nN/μm, this translates to a precision in force of 0.1–0.3 nN.

### Profile microindentation gives similar apparent stiffness values to Atomic Force Microscopy

**Figure 3:**
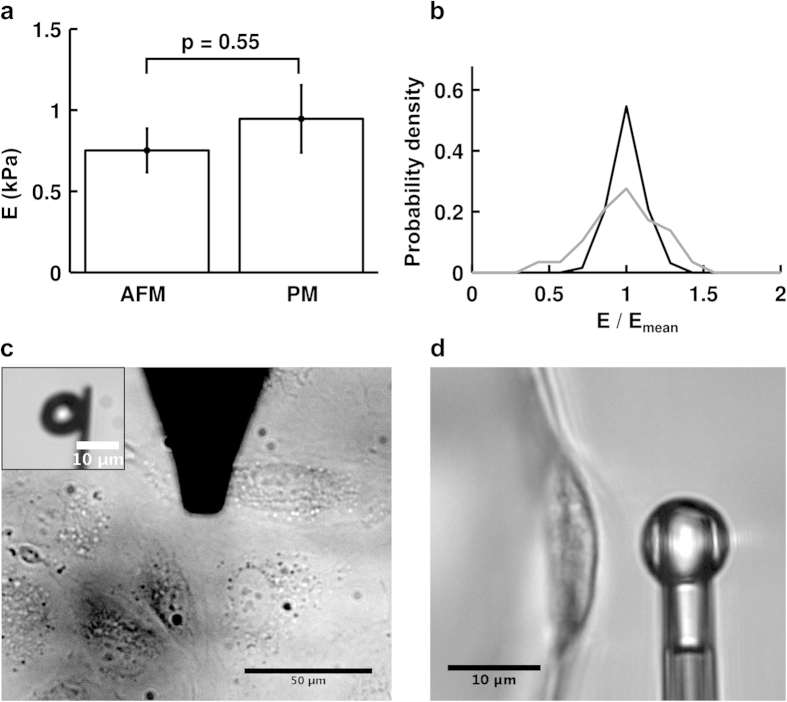
Comparison of apparent stiffness obtained by AFM and profile microindentation. (**a**) The difference between the two means is not statistically significant (p = 0.55; two-tailed Student’s t-test). Data are mean ± s.e.m. n = 5 cells for AFM and n = 10 cells for profile microindentation. (**b**) Probability density for the measure of the apparent stiffness of a given cell normalized by the mean apparent stiffness found for that cell. Black is for AFM and grey for profile microindentation. The same cells as in panel a are used. (**c**) Top view of AFM measurement of HUVEC rigidity. (Inset) Side view of the spherical probe glued to the tip used in the AFM measurement. (**d**) Side view of profile microindentation measurement of HUVEC rigidity.

We compared the apparent stiffness of HUVECs measured using profile microindentation to that obtained via AFM, as this latter method is widely used to measure cell mechanical properties^12^,^13^,^14^,^15^,^16^,^17^,^18^. In both cases, we used an approach speed of 1 μm/s, a spherical indenter of radius 5 μm, indented on top of the nucleus and fit the entire force-deformation curve using a Hertzian model (assuming a Poisson’s ratio of 0.5, see next paragraph for details). The threshold forces for indentation were in the same range, with 3–6 nN for AFM and 5 nN for profile microindentation, and so were the indenter rigidities, with respective values of 11.5 nN/μm and 5.0 nN/μm. The measurements were made at temperatures of ~37 °C in both cases. The substrate was the main difference between the two setups. While HUVECs adhered to glass in the AFM experiment, they adhered to Cytodex-3 dextran beads in the profile microindentation experiment. However, with respective rigidities of ~70 GPa and ~50 kPa, both glass and dextran beads are much stiffer than cells, and previous investigators have shown that while substrate stiffness matters greatly when its rigidity is comparable to that of the cell, this is no longer the case when substrate stiffness is very high compared to the cell^28^. Indeed, the measured apparent stiffnesses were found to be similar using the two different methods (0.75 ± 0.14 kPa for AFM vs. 0.95 ± 0.21 kPa for profile microindentation; p = 0.55) (Fig. 3). Such a comparison validates the profile microindentation technique and positions it as a low-cost complementary approach to more conventional AFM colloidal indentation systems.

The repeatability of the measurements of apparent stiffness was also assessed under these experimental conditions by investigating how the apparent stiffness varied for a given cell during several consecutive measurements. The dispersion of the measurements was found to be about twice as high for profile microindentation compared to AFM, as the standard deviation for the normalized apparent stiffness was 0.1 for AFM and 0.2 for profile microindentation (Fig. 3).

### In profile microindentation stress relaxation experiments, cell mechanical properties can be described by two independent parameters: an apparent stiffness E^*^ and a dimensionless relaxation parameter α

#### The apparent stiffness E*

We first assess the cell rigidity by focusing on the approach phase of the indentation. The force-indentation curve of an adherent cell indented by a spherical indenter is well described by the classical Hertz equation^29^:


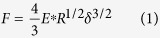


where F is the force, E^*^ the apparent stiffness (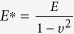, with E the Young’s modulus and υ the Poisson’s ratio), R an effective radius which is a function of the indenter’s radius R_probe_ and the cell apical surface radius of curvature R_cell_ (R = 1/(1/ R_probe_ + 1/ R_cell_)), and δ the indentation depth. In our case, the contact position d_c_ must be determined in order to assess indentation; therefore, we obtained E*, d_c_ and 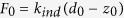 through a fit of the following equation:


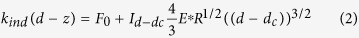


where


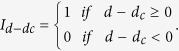


**Figure 4:**
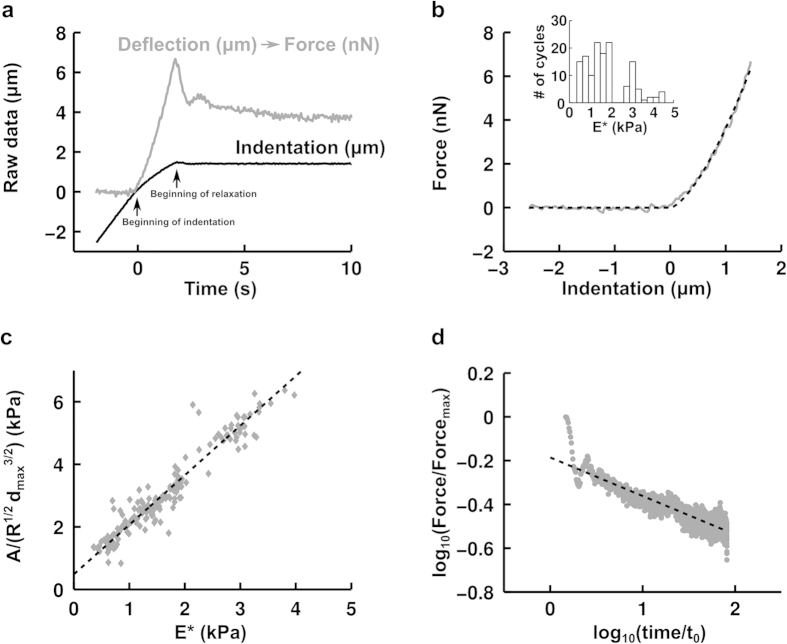
BAEC stress relaxation can be described using only two independent mechanical parameters: the apparent stiffness E* and the relaxation parameter α. (**a**) Example of raw data extracted from cross-correlation image analysis. We obtain the indentation (d − d_c_) (black curve) and the deflection (d − z) − (d_0_ − z_0_) (grey curve). t = 0 s marks the beginning of the indentation (left arrow). By multiplying the deflection by the indenter’s rigidity k_ind_ (nN/μm), we find the applied force F (nN). After the chosen force F_threshold_ is attained (right arrow), we maintain the indentation constant, ensuring constant strain during force relaxation. (**b**) Example force-indentation curve fitted with a single parameter: the apparent stiffness E*. In this example, approach speed is 1.4 μm/s. Data acquisition frequency is approximately 30 Hz. The inset represents a histogram of the apparent stiffness E* of BAECs (n = 20 cells and N = 139 indentation curves) measured with a microindenter whose base is moving at 1.4 μm/s, fitting the first 1.0 μm of the force-indentation curve. (**c**) Scatter plot of A/(R^1/2^d_max_^3/2^) as a function of E*, where A is the pre-factor in the force relaxation as given by equation (3), R is the effective radius given by R = 1/(1/ R_probe_ + 1/ R_cell_) with R_cell_ ~ 20 μm, d_max_ is the indentation maintained during relaxation, and E* is the apparent stiffness measured by fitting the first 1.0 μm of the force-indentation curve of BAECs (n = 51 cells and N = 191 indentation curves). The very good correlation (correlation coefficient r = 0.95) shows that A and E* are inter-dependent variables, both measuring a cell’s apparent stiffness. (**d**) Example force-time relaxation curve at fixed indentation. Cell relaxation is observed over 80 s. Time is normalized by t_0_ = 1 s. Force is normalized by its maximum value attained at the first time point.

The characteristic function 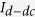 is used to take into account the pre-contact case in which no force is applied on the cell. An example of such a fit for a BAEC can be seen in Fig. 4 and provides the first mechanical parameter: the apparent stiffness E* of the cell. We estimate the quality of the fit by evaluating the square root of the mean of the L2-norm of residuals between the fit and the experimental data. We obtain a value in the example given of 0.14 nN, which is of the same order of magnitude as our precision in force.

In our data analysis protocol, we perform the fitting of the force-indentation curve twice: a first time to get an approximate contact point d_capp_, and a second time where we fit only the data where d 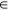 [d_capp_ − 2 μm; d_capp_ + 1 μm]. For an indentation speed of 1.4 μm/s, we find E* = 1.8 ± 0.086 kPa (mean ± s.e.m.) (Fig. 4), in line with values found in the literature^10^,^30^,^31^,^32^,^33^. To obtain the Young’s modulus, one can assume a Poisson’s ratio of 0.5^11^,^34^, corresponding to an incompressible medium, which is best suited when modeling the cell as a homogeneous isotropic medium during moderate indentations. Here, “moderate indentations” denotes indentations in which the applied pressure P_app_ is small compared to the osmotic pressure P_osm_ of isotonic saline which acts to maintain cell volume constant^10^.

In support of this notion, during indentations with a P_app_ on the order of 1 kPa (close to our experimental values, see Fig. 4 with P_app_ ~force/contact area ~7 nN/15 μm^2^ ~0.5 kPa) observed with a confocal microscope, Harris and Charras reported no volume change^4^ (see supplementary discussion for a more detailed discussion of appropriate values of Poisson’s ratio depending on the experiment).

An advantage of the profile microindentation technique is the ability to readily determine the apparent stiffness of non-adherent cells. To do so, we hold the non-adherent cell with a micropipette. To showcase this capability, we measured the mechanical properties of human primary T lymphocyte CD4 cells (Supplementary Fig. S5).

#### The relaxation parameter α

Once the desired indentation d_max_ is attained, we observe force relaxation at fixed indentation (see Methods for details). We find that the force relaxes according to a weak power-law (Fig. 4) following:


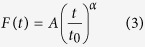


where F is the force, A is a fitting parameter, t is time, t_0_ is an arbitrary time constant which we set at 1 s, and α is another fitting parameter. We observe that there are oscillations in the first ~30 points of our force relaxation curve in Fig. 4 (corresponding to ~1s since data acquisition frequency is ~30 Hz), which are due to our feedback loop that attempts to maintain the position constant^10^.

We note that A is an extensive property. By analogy with the Hertz equation, we normalize A by R^1/2^d_max_^3/2^ to make it an intensive property in order to compare it with the apparent stiffness E* found previously. We find that A and E* are nearly proportional to one another (Fig. 4). This was observed to be true for ~200 indentations performed on ~50 cells at three different approach speeds (1.4 μm/s, 2.8 μm/s and 14 μm/s), using two different indenters, and with and without incubation in cytochalasin-D (a drug that depolymerizes actin filaments and renders cells softer). This indicates that A and E* are inter-dependent variables; henceforth, we choose to retain E*.

### The apparent stiffness E^*^ is indentation depth-dependent, while the relaxation parameter α is not

**Figure 5:**
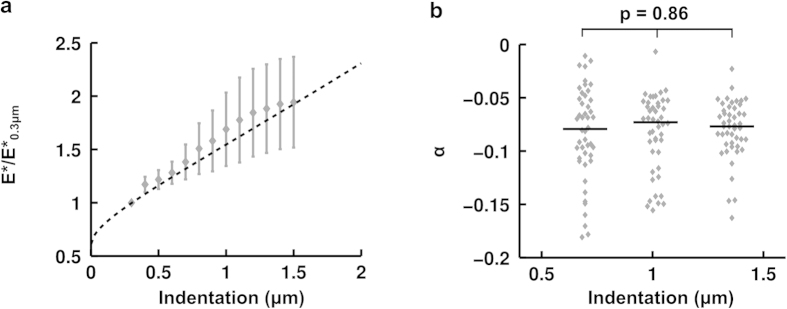
Effect of indentation depth on measured cell mechanical properties. (**a**) Apparent stiffness E* (mean: grey diamonds and S.E.M.: grey bars) of BAECs as a function of the indentation depth used for fitting the data (n = 20 cells). For each curve, E* was normalized by its value at an indentation depth of 0.3 μm. Also shown is the apparent stiffness E* obtained by fitting an analytical force-indentation curve taken from Dimitriadis’ formula that accounts for cell depth (dotted black line)^37^,^38^. The unique fitting parameter, the apparent stiffness E*, was chosen to match experimental data at an indentation depth of 0.3 μm. Data are mean ± s.e.m. (**b**) Relaxation parameter α as a function of indentation depth. Each point (grey diamond) represents an indentation (n = 20 cells with 7 indentations each). Curves were separated into 3 groups sorted according to indentation depth. The x axis represents a group’s mean indentation depth. Each group is represented as a scatter plot (median: black line). A one-way ANOVA with the null hypothesis that all samples are drawn from the same population gives a p-value of 0.86.

In order to test the dependence of the apparent stiffness E* on the indentation length scale, we fitted identical force-indentation curves on intervals of increasing lengths, from d 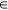
[d_0_ − 2 μm; d_0_ + 0.3 μm] to d 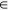
[d_0_ − 2 μm; d_0_ + 1.5 μm], with the function described in equation (2). For each force-indentation curve, the values obtained for the apparent stiffness E* were normalized by those obtained when using the smallest interval [d_0_ − 2 μm; d_0_ + 0.3 μm] (Fig. 5). We find that the cell’s apparent stiffness increases with the depth of indentation.

To test the hypothesis that this increase is due to the effect of the substrate, as has been previously observed^35^,^36^, we fitted equation (2) for varying intervals to a theoretical force-indentation curve obtained using the following equation proposed by Dimitriadis *et al.* that accounts for substrate effects at small depths for an incompressible bonded substrate^37^:


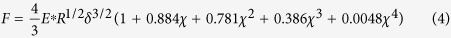


with 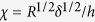, where we took for h the average of the cell heights measured in our experiments (h = 4.5 μm). Again, we normalized the values obtained for E* for various intervals by the value found for a maximum indentation of 0.3 μm, and we find good agreement with our data (Fig. 5).

To further test if the relaxation parameter α was also indentation length scale-dependent, we investigated how it varied with indentation depth and found no significant difference at the depths tested (Fig. 5). We conclude that this parameter does not depend on length scale, as has been reported elsewhere using oscillating beads^23^ or creep relaxation^19^.

### The apparent stiffness E* depends on indentation speed through the duration of the indentation

**Figure 6:**
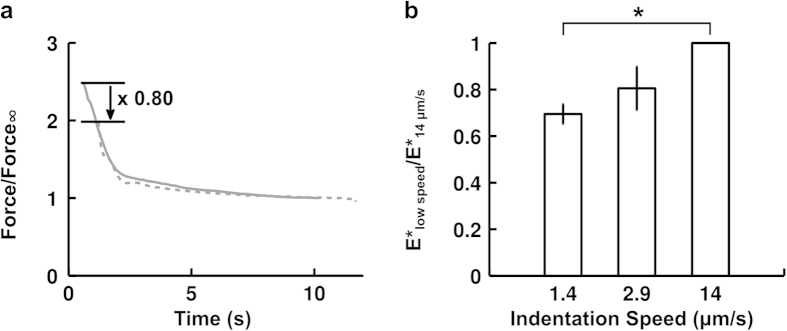
Effect of indentation speed on force relaxation and apparent stiffness. (**a**) Average force relaxation curve at fixed indentation for fast (n = 13 cells, grey line, indentation speed 14 μm/s) and slow (n = 20 cells, dotted grey line, indentation speed 1.4 μm/s) indentations. Force is normalized by its value after 10 s of relaxation for each cell. Time on the x axis starts at the beginning of indentation. (**b**) Comparison of cells’ apparent stiffness as a function of indentation speed. For each cell, its apparent stiffness at low speed was normalized by its value at 14 μm/s. Data are mean ± s.e.m. n = 5 cells for 1.4 μm/s and n = 3 cells for 2.9 μm/s. The p-value from a paired two-tailed Student’s t-test with the null hypothesis being that the ratio is equal to 1 is significant for 1.4 μm/s (p = 0.002) but not for 2.9 μm/s (p = 0.171). An indentation speed of 2.9 μm/s means that the full indentation will last approximately ~1 s.

To test the dependence of our mechanical parameters E* and α on indentation speed, we compared the relaxation profiles at the two indenter speeds of 1.4 and 14 μm/s (Fig. 6). Each force relaxation curve, obtained at a fixed indentation, was renormalized by its value after 10 s of relaxation. All the curves at a given speed were then averaged. When the reference time t = 0 s is taken to be the beginning of the indentation, we find rather good agreement between the two averaged relaxation curves, which collapse on a master curve. It thus follows, as can be seen in Fig. 6, that the slower the indentation, the longer a cell will have to relax and hence the softer it will appear.

### Tracking the evolution of cell mechanical properties upon “whiffing” a drug onto a cell

Because we measure cell mechanical properties in ~10 s, we are able to repeat that measurement to determine how the mechanical properties of a particular cell evolve in response to an external stimulus applied locally to that cell. To demonstrate this capability, we used a micropipette to “whiff” cytochalasin-D onto a BAEC, as depicted in Fig. 1. The mean “whiffing” fluid velocity is ~10 cm/s; thus, cytochalasin-D convection dominates diffusion (characteristic convection time τ_convection_ ~10^−3^ s ≪ τ_diffusion_ ~10 s; see supplementary discussion for details). We performed measurements of mechanical properties every 30 s for a period of ∼40 min. We compared our “whiffing” experiment to two other cases: a control case with no “whiffing” to verify that the mechanical measurements were not disruptive to the cell, and a case where the cells were continuously incubated in cytochalasin-D to see how effective “whiffing” a drug at a given concentration is compared to a more standard incubation protocol.

**Figure 7:**
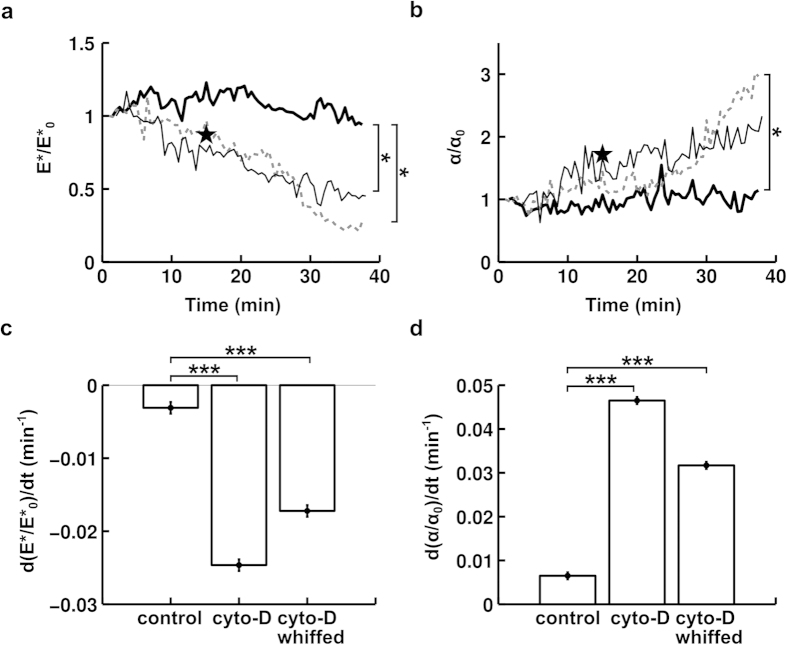
Impact of cytochalasin-D on BAEC mechanical properties. (**a**) Time evolution of BAEC apparent stiffness E*. E* is normalized by its value at t = 0 min E*_0_. The indentation speed is 1.4 μm/s. Thick black line is control (n = 7 cells), grey dotted line is for cells incubated in cytochalasin-D at 500 nM starting at t = 5 min (n = 9 cells), and thin black line is for cells that were “whiffed” with cytochalasin-D at 500 nM starting at t = 5 min (n = 5 cells). Large black pentagram positioned at t = 20 min represents a separate experiment where control cells’ apparent stiffness, E*_control_ (n = 13 cells), was compared to that of cells incubated for 15 min in cytochalasin-D at 1000 nM, E*_cyto-D_ (n = 18 cells). The y-coordinate of the pentagram is E*_cyto-D_/E*_control_. (**b**) Time evolution of BAEC relaxation parameter α. The same notation as in panel a is used. (**c**) Time derivative of data in panel a: left column is control case, middle column represents cells incubated in cytochalasin-D, right column represents cells “whiffed” with cytochalasin-D. Data are mean ± s.e.m. Slopes in panel a were compared using the ANOCOVA test. *** indicates p < 0.001. (**d**) Time derivative of data in panel b. Data are mean ± s.e.m. The same notation as in panel c is used.

As can be seen in Fig. 7, the cell’s apparent stiffness E* decreases by ~50–70% over the duration of the experiment, in line with values found in the literature. In addition to becoming softer upon initial indentation, the cell also relaxes faster, as indicated by a 2–3 fold increase in the relaxation parameter α over the duration of the experiment. Performing repeated indentations provides the advantage of directly observing the kinetics of a drug’s activity on the cell. Here, we see that the rates of both the decrease in apparent stiffness and the increase in the relaxation parameter are relatively constant in time.

To compare cytochalasin-D action kinetics quantitatively, we compared the slopes of the time evolution of the mechanical properties using an ANOCOVA test. This allows greater statistical robustness (p < 0.001, see Fig. 7) than comparing cases at selected time points, thus partially overcoming the drawback of this technique’s low throughput. We find that, while the mechanical properties barely change in the control case, they evolve dramatically and qualitatively similarly in the two other cases. We note that with our choice of indentation duration (~1 s), the normalized apparent stiffness decreases nearly proportionally to the fluidization of the cell, as we have in all three cases |d(α/α_0_)/dt| ~2 |d(E*/E*_0_)/dt|.

**Figure 8:**
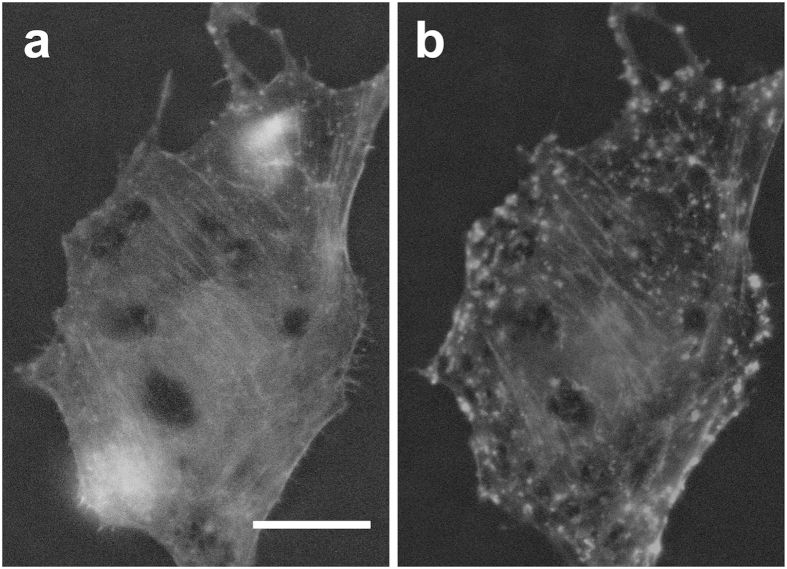
Effect of cytochalasin-D on BAEC actin filaments. (**a**) Control (untreated) BAEC. (**b**) Same cell as in panel a after incubation for 120 min in cytochalasin-D at 1000 nM. Note actin filament bundles being disrupted at various locations throughout the cell and actin aggregates forming at the cell periphery. Scale bar is 20 μm. See supplemental movies S2 and S3 for time lapse of actin filament depolymerization over time under the effect of cytochalasin-D.

To confirm that cytochalasin-D had the intended effect of disrupting actin filaments, we used live-cell fluorescence imaging (LifeAct) to visualize actin filaments over time during incubation in cytochalasin-D (Fig. 8 and supplementary movies). Most actin filaments progressively depolymerize and form small bundles (bead-like structures in Fig. 8).

Beyond simply “whiffing” a drug onto a cell, the second micropipette used for injecting cytochalasin-D above can also be used to bring in another cell and study cell-cell interactions and the effects of these interactions on mechanical properties. To demonstrate this capability, we used a micropipette to place human lymphoblast cells on human aortic endothelial cells (HAEC) and observed in profile view as the lymphoblast migrated on the endothelial cell surface (Supplementary Fig. S6), all the while measuring the mechanical properties of the endothelial cell (data not shown).

## Discussion

The scale-free power law found using profile microindentation is analogous to the one identified in creep relaxation and bead oscillation experiments. Indeed, the power-law behavior observed here has previously been reported, notably in creep relaxation^19^ and in bead oscillation experiments where several orders of magnitude of frequencies were sampled^23^. Following a calculation performed by Balland *et al.*^39^, we show in what follows that although our experiment is performed at constant strain rather than constant stress and in the time domain rather than the frequency domain, these approaches are equivalent.

By analogy with our stress relaxation function 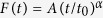, we introduce the creep relaxation function 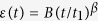 (strain evolution under constant stress) for an elastic body with identical mechanical properties. Following some mathematical derivations (see supplementary discussion for details), we find that 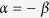 and 
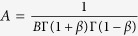

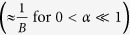
, where Γ is the Euler function. Thus, the power-law exponent we find for our constant strain experiment can be compared to previous work reported in the literature for creep relaxation and bead oscillations experiments simply by changing the sign.

The current results also show that the pre-factor A in the stress relaxation law 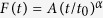 and the apparent stiffness E* measured during the cell indentation ramp-up are inter-dependent (Fig. 4); therefore, E* and the relaxation parameter α are sufficient to describe cell indentation and subsequent relaxation.

In order to fit the force-indentation curve during the approach phase, we have used a non-adhesive contact model because the lack of measurable negative force indicates that the adhesive forces are small during this phase (see Fig. 4). This is not surprising because the ions present in the medium (DMEM) screen electrostatic interactions. The medium is further supplemented with 10% serum, and hence contains a large amount of bovine serum albumin (BSA), which has a well-known anti-adhesive effect. The non-adhesive contact assumption, however, is not expected to be valid after contact has been made during the retraction phase where, for example, an adhesive force F_ad_ = 0.14 F_max_ (F_max_ is the maximum force) is measured for the BAECs in the inset of Fig. 4b. In this case, we can use the resulting adhesion energy per unit surface area defined as γ = −F_ad_/(3πR_probe_) = 13 μN/m to compute the dimensionless parameter λ proposed by Maugis^40^
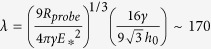
, where h_0_ is the equilibrium separation between the surfaces, typically taken to be 0.4 nm for solids. Because λ ≫ 5, the Johnson-Kendall-Roberts (JKR) model would apply during the retraction phase (not treated here).

Further, we observe that cell relaxation curves are self-similar if we consider the beginning of indentation as the initial point. In our cell indentation experiments, cell relaxation appeared independent of indentation speed (from 1.4 to 14 μm/s) and exhibited a self-similar behavior when the beginning of indentation was taken as the initial time point, i.e. t = 0 s (Fig. 6). This means that if one sets the origin of time for relaxation not at the beginning of relaxation per se but rather at the moment where mechanical energy is injected into the system, the force relaxation curves of two groups of cells (n = 20 cells and n = 13 cells) indented at very different speeds (1.4 μm/s and 14 μm/s respectively) collapse on a master curve. This finding suggests that cell relaxation is driven by the time at which an external energy input initializes the system. We therefore propose that this time point is more relevant to study cell relaxation than the beginning of cell relaxation, which corresponds to the end of the indentation phase.

Finally, by performing 10 second-long profile microindentations every 30 s for more than 30 min, we were able to monitor the viscoelastic properties of endothelial cells almost continuously for an extended period of time (Fig. 7). In the control case, cell viscoelastic properties remained nearly constant over a period of time longer than 30 min (Fig. 7), indicating that the measurement technique itself is minimally disruptive to the cell.

To demonstrate our ability to act on a single cell’s local environment and to evaluate the impact of a local external stimulus on the cell’s mechanical properties dynamically, we “whiffed” cytochalasin-D continuously onto a single BAEC (Fig. 1) and monitored the evolution of cell mechanical properties over time using our profile microindentation technique. We chose cytochalasin-D because its effect on actin filaments is well documented and it has been reported to soften cells^4^,^23^ and, perhaps less predictably, to render adherent cells more fluid-like^23^ (interestingly, non-adherent cells, such as neutrophils, have been reported to soften but to become more solid-like^41^). Consistent with these previous studies, we find that adherent endothelial cells’ apparent stiffness E* decreases over time (Fig. 7), and that the absolute value of their relaxation parameter α (α has a negative sign) increases over time (Fig. 7). Indeed, as discussed in Fabry *et al.*^23^, a value of the relaxation parameter α close to 0 indicates solid-like behavior, while a value closer to −1 (and therefore with an increased absolute value) indicates fluid-like behavior (to compare to the quantity termed 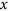 in the cited work, one needs to recognize that 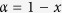). Further, we observe that at a given concentration, cytochalasin-D affects a cell’s mechanical properties in a near-continuous fashion. Finally, we note that at indentation durations of ~1 s, the reduction in normalized apparent stiffness goes together with the fluidization of the cell, suggesting the same origin for both mechanical properties, presumably here the cytoskeleton as it is the primary component of the cell affected by cytochalasin-D (Fig. 8).

These results demonstrate our ability to “whiff” a drug, in this case cytochalasin-D, at a well defined location and at selected time points and to simultaneously use profile microindentation to monitor the evolution of a cell’s viscoelastic properties.

## Conclusion

We demonstrate the ability of the profile microindentation technique to measure mechanical properties of both adherent and non-adherent cells. Using our profile microindentation technique, we show that an adherent cell’s indentation and relaxation under constant strain can be characterized using only two mechanical parameters, the apparent stiffness E* and a relaxation parameter α. While the apparent stiffness E* depends on both indentation depth and speed, the relaxation parameter α is scale-free and is identical (with a minus sign) to the exponent in a weak power-law describing force relaxation found by other investigators using, for instance, bead oscillation^23^ or creep relaxation^19^ experiments. The apparent stiffness measured using profile microindentation matches that found using AFM, validating the approach.

Importantly, the profile microindentation technique offers the capability of easily adding a micropipette to the setup, which gives us the ability to test drugs by “whiffing” them onto a cell, at a controlled location and time, without introducing mechanical perturbation of the setup stability which is often challenging in AFM experiments. This makes this technique well suited to investigate the effect of a convective flux on a single-cell, to determine for instance if drug intake kinetics are impacted by fluid velocity when a drug is administered via convection-enhanced delivery, or if fluid shear stress in itself would impact a cell’s physiology. In future investigations, the micropipette could be used to locally introduce an agonist or to bring another cell in contact with the cell whose mechanical properties are being measured and thus explore the effect of cell-cell contact on cell mechanics.

## Additional Information

**How to cite this article**: Guillou, L. *et al.* Dynamic monitoring of cell mechanical properties using profile microindentation. *Sci. Rep.* **6**, 21529; doi: 10.1038/srep21529 (2016).

## Supplementary Material

Supplementary Information

Supplementary Movie S1

Supplementary Movie S2

Supplementary Movie S3
