# Supplementary material for: Dynamic monitoring of cell mechanical properties using profile microindentation
Source: Sci Rep. 2016 Feb 9;6:21529. doi: 10.1038/srep21529 (PMC4746699; doi:10.1038/srep21529)
Supplement: Supplementary Information [file srep21529-s1.pdf]

Dynamic monitoring of cell mechanical properties using profile microindentation

L. Guillou[1], A. Babataheri[1], P.-H. Puech[2,3,4], A. I. Barakat[1], J. Husson[1]*

[1] Hydrodynamics Laboratory (LadHyX), Department of Mechanics, Ecole Polytechnique, 91128 Palaiseau, France

[2] Aix Marseille University, LAI UM 61, Marseille, F-13288, France

[3] Inserm, UMR_S 1067, Marseille, F-13288, France

[4] CNRS, UMR 7333, Marseille, F-13288, France

*Correspondence: julien.husson@ladhyx.polytechnique.fr

Supplementary Information

Supplementary Movie S1 (GuillouS1_supplementary_BAEC_indentation_demo.avi): Supplemental movie showing a profile microindentation of a BAEC. The acquisition was performed at 1 frame per second, and the movie plays at 5 frames per second (i.e., time accelerated 5 times).

Supplementary Movie S2 (GuillouS2_supplementary_BAEC_cytoD_1000nM_120min.avi): BAECs were incubated in cell culture medium containing cytochalasin-D at 1000 nM and observed under fluorescence microscopy in order to visualize actin filament depolymerization over time. Time is accelerated 300 times (duration of movie: 24 s, duration of experiment: 120 min).

Supplementary Movie S3 (GuillouS3_supplementary_BAEC_cytoD_500nM_120min.avi): BAECs were incubated in cell culture medium containing cytochalasin-D at 500 nM and observed under fluorescence microscopy in order to visualize the kinetics of actin filament depolymerization. Time is accelerated 300 times (duration of movie: 24 s, duration of experiment: 120 min).

Supplementary discussion

Equivalence between time and frequency domain and between creep and relaxation experiments

In the linear elastic regime, stress and strain are related as follows:

$$\sigma(t) = K(t)\varepsilon_0 + \int_0^t K(t-t')\dot{\varepsilon}(t')dt' \qquad (1)$$

where σ is the stress, t the time, K the stress relaxation function at fixed strain, ε_0 the strain at t=0, and $\dot{\varepsilon}$ the strain rate. This translates in the Laplace domain to

$$\tilde{\sigma}(s) = s\tilde{K}(s)\tilde{\varepsilon}(s) \qquad (2)$$

where the tilde denotes the Laplace transform. For a sinusoidal excitation $\varepsilon(t) = \varepsilon(w)e^{jwt}$ (with w the pulsation), we have $\sigma(t) = \sigma(w)e^{jwt}$ and the complex viscoelastic modulus G is defined by $G(w) = \sigma(w)/\varepsilon(w)$. In our experiment, $K(t) = A(t/t_0)^{\alpha}$, which translates in the Laplace domain to $\tilde{K}(s) = A\frac{\Gamma(1+\alpha)}{s(st_0)^{\alpha}}$, where Γ is the Euler function and $\Gamma(1+\alpha) = \int_0^{+\infty} exp(-x)x^{\alpha}dx$. Comparing to the result obtained for the creep function $J(t) = B(t/t_1)^{\beta}$ in supplementary reference 1, we conclude that:

$$\alpha = -\beta \qquad (3)$$

and $A = \frac{1}{B\Gamma(1+\beta)(1-\beta)}$ $\left(\approx \frac{1}{B} \text{ for } 0 < \alpha << 1\right)$ (4)

Equation (3) implies that the power-law exponent for a constant strain experiment is the opposite of that of creep function experiments and oscillating bead trapping [2]. We note that according to Fabry et al. [3], the exponent α provides access to the loss tangent η at low frequencies (η = απ/2), which in turn provides access to the loss modulus, knowing the

storage modulus (by definition, η = G''/G', where G'' is the loss modulus and G' the storage modulus).

Which value of a cell's Poisson's ratio for which experiment?

When the Poisson's ratio retains its original definition as one of two elastic moduli necessary to describe a homogeneous isotropic elastic medium, one should take a value of 0.5 for moderate cell compressions, indicating that cell volume is conserved. Indeed, Harris and Charras reported no volume change [4] when observing cell indentation with a confocal microscope for forces on the order of 10 nN corresponding to strains on the order of 10 to 40%. To the best of our knowledge, only in cases where large compressions are applied over long durations (deformations on the order of 100% over 300 s as in reference 5) has overall cell volume change due to compression been reported. It is believed that the volume change then results from the efflux of fluid through the cell membrane, which means that a model of a cell as a continuous elastic medium is ill-defined in the first place, as a portion of the medium is "lost". Other values of Poisson's ratio that have been reported when modeling the cell as an elastic medium may have been the result of either indirect measurements [6] or have resulted from approximating the cell as a two-dimensional medium [7].

In some studies, investigators have sometimes used the term Poisson's ratio to refer to the Poisson's ratio in a biphasic or poroelastic model, which is a physical model distinct from the elastic medium. In the poroelastic model, what is sometimes referred to as the Poisson's ratio is strictly speaking the drained Poisson's ratio [8], also called the solid-phase Poisson's ratio, and refers to the compressibility of the solid-phase. For this solid-phase Poisson's ratio, typical values of 0.3-0.4 have been reported for the solid matrix in cells [5,9,10].

Convection dominates diffusion in "whiffing" experiment

In the present study, "whiffing" is accomplished by using a syringe to pressure an air reservoir that in turn pushes on the fluid containing the agent being "whiffed". For laminar

flow (which we will verify at the end of the analysis) through a circular cross-section duct, Poiseuille's law [11] relates the average flow velocity and the applied pressure difference as: $v = \frac{\Delta P R^2}{8\eta L}$, where v is the mean fluid velocity, ΔP is the applied pressure difference, R is the radius of the cylindrical duct, η is the fluid dynamic viscosity, and L is the length of the duct. Because the hydraulic resistance R_h (defined as $R_h = \Delta P/Q$) in a Poiseuille flow scales as $1/R^4$, a good approximation of the overall micropipette resistance can be obtained by taking the resistance of the thin tip, where the radius is ~10^{-5} m, and neglecting the long shaft, where the radius is ~10^{-3} m.

Given an applied pressure difference ΔP ~1 kPa, a micropipette radius R ~10^{-5} m, a micropipette length L ~10^{-3} m, a dynamic viscosity η ~10^{-3} Pa.s (water at room temperature), we find an approximate mean velocity of 10^{-1} m/s. This leads to a Reynolds number close to the tip of the micropipette of $\mathrm{Re} = \frac{\rho v R}{\eta}$ ~1 (ρ is the density of water), justifying our initial assumption of a laminar flow. The distance between the micropipette tip and the cell is L ~10^{-4} m, which leads to a characteristic time for convection of cytochalasin-D of $\tau_{convection}$ ~10^{-3} s. The diffusion time scales as L^2/D, where D is the diffusion coefficient. For spherical particles in a low-Reynolds number flow, D can be obtained from the Stokes-Einstein relation [12] $D = \frac{k_B T}{6\pi\eta R_{part}}$, where k_B is the Boltzmann's constant, T is the absolute temperature, and R_{part} is the radius of the particle. At room temperature, estimating that R_{part} ~10^{-9} m for cytochalasin-D (molar mass ~500 g/mol), we find a characteristic diffusion time constant of $\tau_{diffusion}$ ~10 s.

Comparison of profile microindentation at room temperature vs. physiological temperature

We built a custom-made Plexiglas chamber that allows passage for micropipettes while thermally insulating the Petri dish. Briefly, the chamber was heated with a resistance, where the electrical current was controlled by a PID system branched to a thermocouple device placed inside the chamber. We then calibrated our system to link the target temperature given to the PID to the medium temperature inside the Petri dish. Using this system, we were able to verify that cell indentations could be performed at physiological temperature without increasing the indenter vibrations too much. We find that cell stiffness increases by ~50% (Supplementary Fig. S4) when going from physiological temperature (37 °C) to room temperature (~20 °C), although this difference was not statistically significant. Surprisingly, past studies have shown both an increase [13] and a reduction [14] in epithelial cell stiffness for this temperature variation.

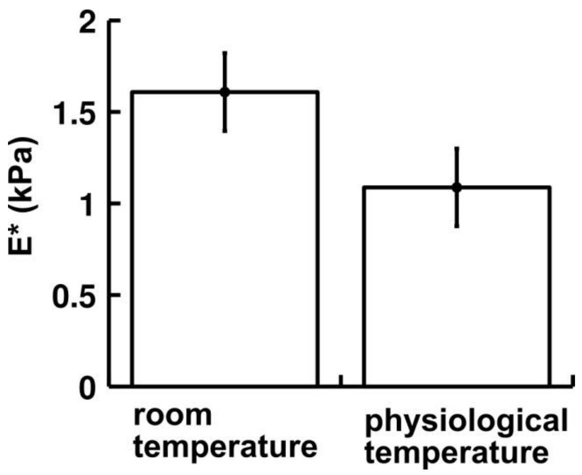

SUPPLEMENTARY FIGURE S4 Effect of temperature on cell apparent stiffness. Indentation speed was 1.4 µm/s. Left column represents cells indented at room temperature, while right column represents cells indented at physiological temperature. Data is mean ± SEM. Difference is not statistically significant (p = 0.08).

Profile microindentation may also be used on non-adherent cells

To demonstrate the capability of using profile microindentation to determine the mechanical properties of non-adherent cells, we performed microindentations of human T lymphocyte CD4 cells that were held with a micropipette (Supplementary Fig. S5b). CD4 cells, kindly provided by C. Hivroz (Curie Institute, Paris, France), were maintained in RPMI medium with 10% SVF and 1% Hepes at 37°C and 5% CO_2 and were prepared according to the protocol in Larghi et al. [15]. This study was conducted according to the Helsinki Declaration, with informed consent obtained from the blood donors, as requested by the Etablissement Francais du Sang. The indenter used had a rigidity of 1.2 nN/µm and a radius of 4 µm. The radius of each CD4 cell was measured to obtain the effective radius used in the Hertzian model.

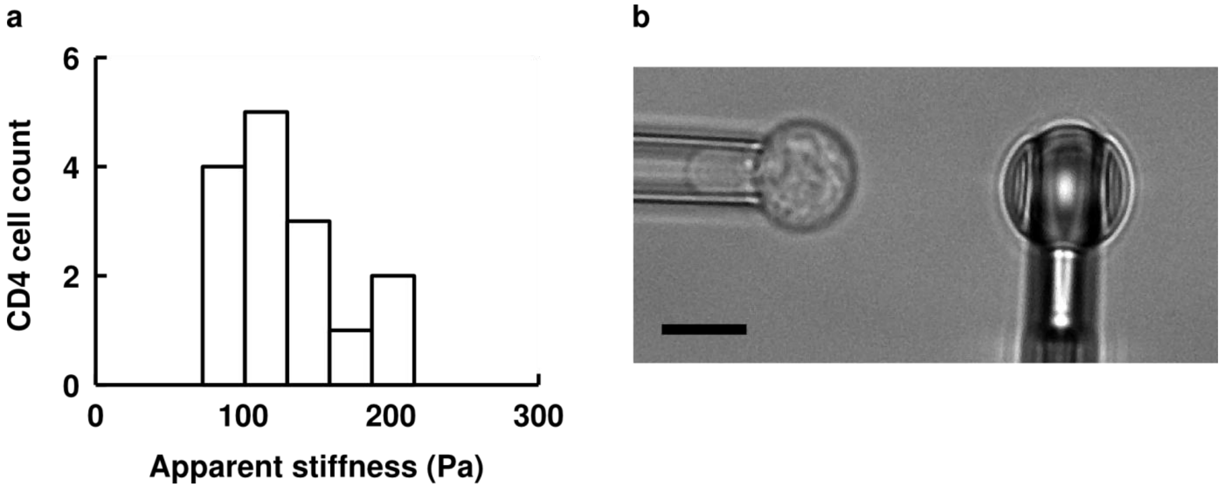

SUPPLEMENTARY FIGURE S5 Profile microindentation of non-adherent human T lymphocyte CD4 cells. (a) Histogram of the apparent stiffness of CD4 cells indented at 0.8 µm/s. (d) Micrograph of a profile microindentation of a CD4 cell. Scale bar is 5 µm.

We found an apparent stiffness of 130 ± 11 Pa (mean ± s.e.m.) (Supplementary Fig. S5a) using an indentation speed of 0.8 µm/s. Values reported in the literature for naïve CD4 cells range from 85 Pa after complete relaxation [16] to 250-300 Pa for indentation speeds between 0.1 and 1 µm/s [17]. The setup and program used were the same as for microindentations of

adherent cells, rendering comparisons easier then when different methods are used (typically micropipette aspiration for non-adherent cells and AFM for adherent cells).

Migration of a human lymphoblast cell on an endothelial cell in profile view

Profile microindentation may be combined with a second micropipette to observe, for instance, leukocytes migrating on endothelial cells. Supplementary Fig. S6 illustrates that the micromanipulation of a human lymphoblast cell does not hinder its ability to migrate on a human aortic endothelial cell (HAEC). All the while, we may perform profile microindentation to monitor the evolution of the mechanical properties of the HAEC (data not shown).

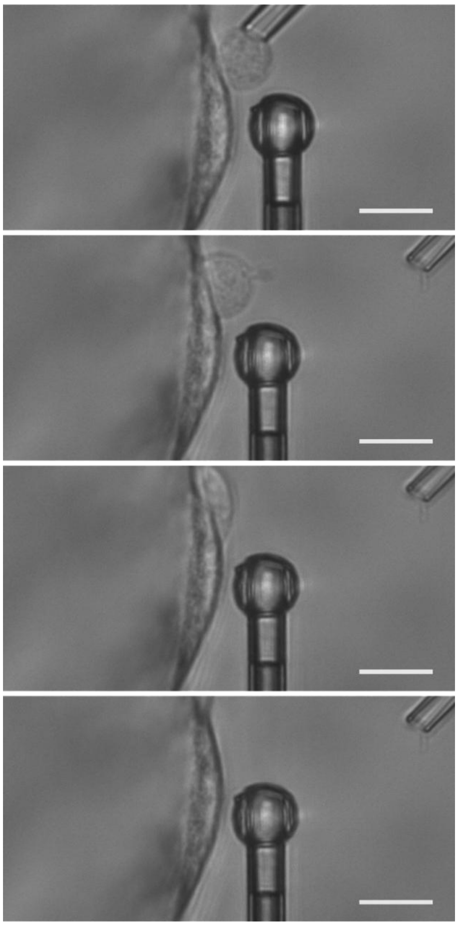

SUPPLEMENTARY FIGURE S6   Profile view of the migration of a human lymphoblast cell on a HAEC. The mechanical properties of the HAEC may be monitored using a microindenter. Scale bar is 10 µm.

In these experiments, human lymphoblast cells were isolated from donor blood about 2 weeks before the experiment by S. Dogniaux and M. Saitakis (Curie Institute, Paris, France) according to the protocol described in Bufi et al. [16]. The cells were maintained in RPMI 1640 medium, Glutamax Supplement GIBCO (Life Technologies # 61870-010) with 1% penicillin-streptomycin GIBCO (10,000 U/mL) (Life Technologies # 15140-122), 1% Hepes GIBCO 1M (Life Technologies # 15630-056), 0.1% 2-Mercaepthanol (50 mM) GIBCO (Life Technologies # 31350-010) and 10% FCS.

Supplementary References

1. Balland, M. *et al.*, Power laws in microrheology experiments on living cells: comparative analysis and modeling. *Phys. Rev. E* **74**, 021911 (2006).
2. Desprat, N., Richert, A., Simeon, J. & Asnacios, A., Creep function of a single living cell. *Biophys. J.* **88**, 2224-2233 (2005).
3. Fabry, B. *et al.*, Scaling the microrheology of living cells. *Phys. Rev. Lett.* **87** (14), 148102 (2001).
4. Harris, A. R. & Charras, G. T., Experimental validation of atomic force microscopy-based cell elasticity measurements. *Nanotechnology* **22** (34), 345102 (2011).
5. Trickey, W. R., Baaijens, F. P. T., Laursen, T. A., Alexopoulos, L. G. & Guilak, F., Determination of the Poisson's ratio of the cell: recovery properties of chondrocytes after release from complete micropipette

aspiration. *J. Biomech.* **39**, 78-87 (2006).

6. Ma, G., Petersen, E., Leong, K. W. & Liao, K., Mechanical behavior of human embryonic stem cell pellet under unconfined compression. *Biomech. Model. Mechanobiol.* **11** (5), 703-714 (2012).

7. Maniotis, A. J., Chen, C. S. & Ingber, D. E., Demonstration of mechanical connections between integrins, cytoskeletal filaments, and nucleoplasm that stabilize nuclear structure. *Proc. Natl. Acad. Sci. U. S. A.* **94**, 849-854 (1997).

8. Detournay, E. & Cheng, A. H.-D., in *Comprehensive rock engineering: principles, practice and projects, vol. II, analysis and design method* (ed. C. Fairhurst, Pergamon Press, 1993), pp. 113-171.

9. Moeendarbary, E. *et al.*, The cytoplasm of living cells behaves as a poroelastic material. *Nat. Mater.* **12**, 253-261 (2013).

10. Shin, D. & Athanasiou, K., Cytoindentation for obtaining cell biomechanical properties. *J. Orthop. Res.* **17**, 880-890 (1999).

11. Poiseuille, J. M., *Recherches experimentales sur le mouvement des liquides dans les tubes de tres-petits diametres* (Imprimerie Royale, 1844).

12. Einstein, A., *Investigations on the theory of the brownian movement* (Courier Dover Publications, 1956).

13. Petersen, N., McConnaughey, W. & Elson, E., Dependence of locally measured cellular deformability on position on the cell, temperature, and cytochalasin B. *Proc. Natl. Acad. Sci USA* **79**, 5327-5331 (1982).

14. Sunyer, R., Trepat, X., Fredberg, J. J., Farre, R. & Navajas, D., The temperature dependence of cell mechanics measured by atomic force microscopy. *Physical Biology* **6**, 025009 (2009).

15. Larghi P,. W. D. J. . C. J.-M. . D. S. . C. K. . B. A. . D. L. . G. K. . G. T. . H.

C., VAMP7 controls T cell activation by regulating the recruitment and phosphorylation of vesicular Lat at TCR-activation sites. *Nature Immunology* **14**, 723-731 (2013).

16. Bufi, N. *et al.*, Human primary immune cells exhibit distinct mechanical properties that are modified by inflammation. *Biophysical Journal* **108**, 2181-2190 (2015).

17. Chang, I. Y.-T., *Study of T cell activation and migration at the single-cell and single-molecule level (PhD thesis)* (Massachusetts Institute of Technology, Dept. of Materials Science and Engineering, 2011).
